# Supplementary material for: The Anti-Inflammatory Effect of Bovine Bone-Gelatin-Derived Peptides in LPS-Induced RAW264.7 Macrophages Cells and Dextran Sulfate Sodium-Induced C57BL/6 Mice
Source: Nutrients. 2022 Apr 1;14(7):1479. doi: 10.3390/nu14071479 (PMC9003490; doi:10.3390/nu14071479)
Supplement: Supplementary file 1 [file nutrients-14-01479-s001.zip › nutrients-1643761-supplementary.pdf]

The anti-inflammatory effect of bovine bone collagen derived peptides in LPS-induced RAW264.7 cells and dextran sulfate sodium-induced C57BL/6 mice

Lujuan Xing[a], Lijuan Fu[a], Songmin Cao[b], Yantao Yin[a], Lanlan Wei[c], Wangang Zhang[a]*

[a] *Key Laboratory of Meat Processing and Quality Control, MOE; Jiangsu Synergetic Innovation Center of Meat Processing and Quality Control; Nanjing Agricultural University; Nanjing, 210095, P.R. China;* Contact information: wangang.zhang@njau.edu.cn

[b] *School of Food and Wine; Ningxia University; Yinchuan, 750021, P.R. China*

[c] *College of Food Engineering, Anhui Science and Technology University; Fengyang, 233100, P.R. China*

Table S1 The composition of peptides in GP

Shown in the EXCLE

Table S2 Primer sequences used in RT-PCR assay

Gene	Forward primer (5'-3')	Reverse primer (5'-3')
GAPDH	ATCACTGCCACCCAGAAG	TCCACGACGGACACATTG
TNF-α	CATCTTCTCAAAATTCGAGTGACAA	TGGGAGTAGACAAGGTACAACCC
IL-1β	TCAAATCTCGCAGCAGCACATC-3′	CCAGCAGGTTATCATCATCATCCC
COX-2	CCCATTAGCAGCCAGTTGTC	CAGGATGCAGTGCTGAGTTC
MCP-1	AACTGCATCTGCCCTAAGGT	CTGTCACACTGGTCACTCCT
TLR-4	GCTCTCAGCCATCCACAAAG	GAGTCGGGAAGAGGAAGAGG
ZO-1	TGAGTGCGTTTCTCTCCCTT	CCCTCTGTGTTCCTCATGGT
Occludin	AGCACTTAACCTGCCTGGAT	AGCCTGTGGAAGCAAGAGAT

Table S3. The scoring criteria for histopathological values

Score	Inflammation	Crypt damage	Ulceration	Edema
0	No infiltrate	None	None	None
1	Occasional cell limited to submucosa	Some crypt damage, spaces between crypts	Small, focal ulcers	Absent
2	Significant presence of inflammatory cells in submucosa, limited to focal areas	Larger spaces between crypts, loss of goblet cells, some shortening of crypts	Frequent small ulcers	Present
3	Infiltrate present in both submucosa and lamina propria, limited to focal areas	Large areas without crypts, surrounded by normal crypts	Large areas lacking surface epithelium	
4	Large amount of infiltrate in submucosa, lamina propria and surrounding blood vessels, covering large areas of mucosa	No crypts		
5	Transmural inflammation (mucosa to muscularis)			

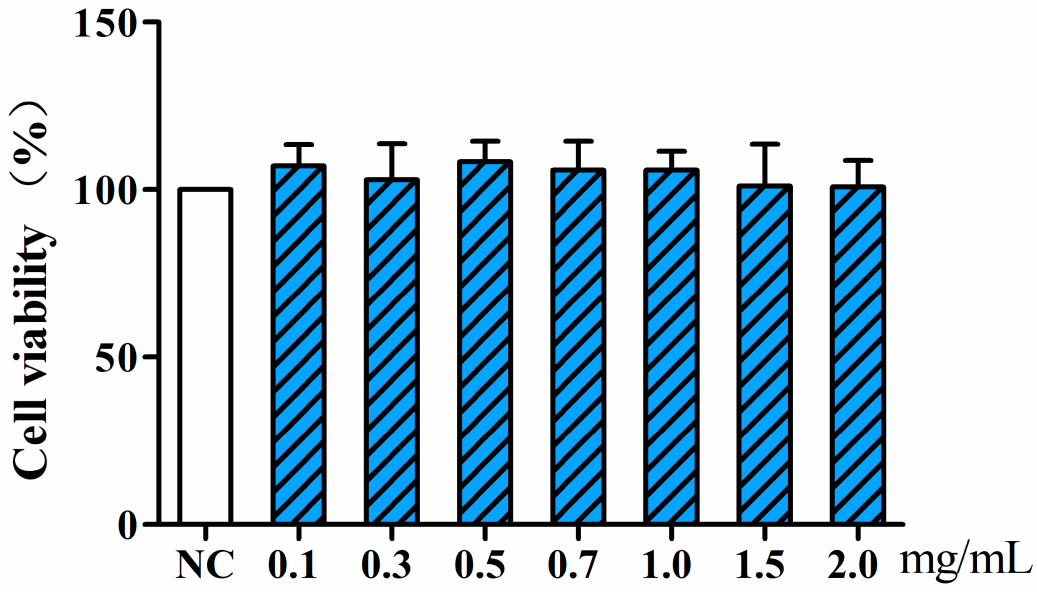

Figure S1. Effects of the GP in RAW264.7 cell viability.

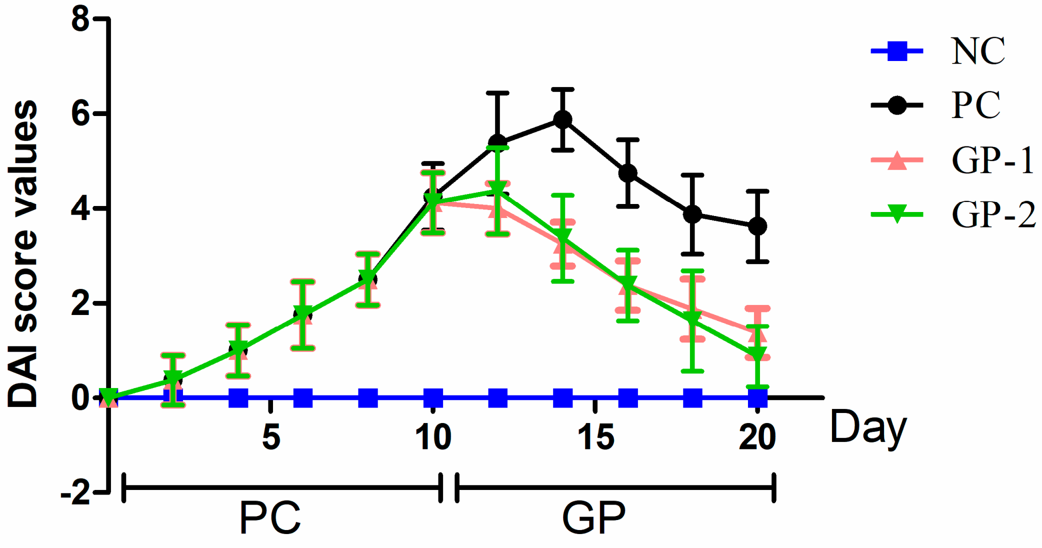

Figure S2.The DAI scores of mice in different groups.

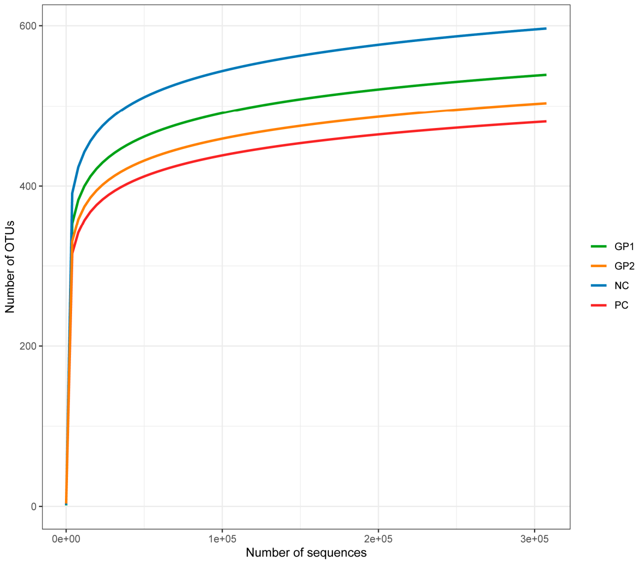
600
400
200
0
Number of OTUs
0e+00
1e+05
2e+05
3e+05
Number of sequences
GP1
GP2
NC
PC

（A）

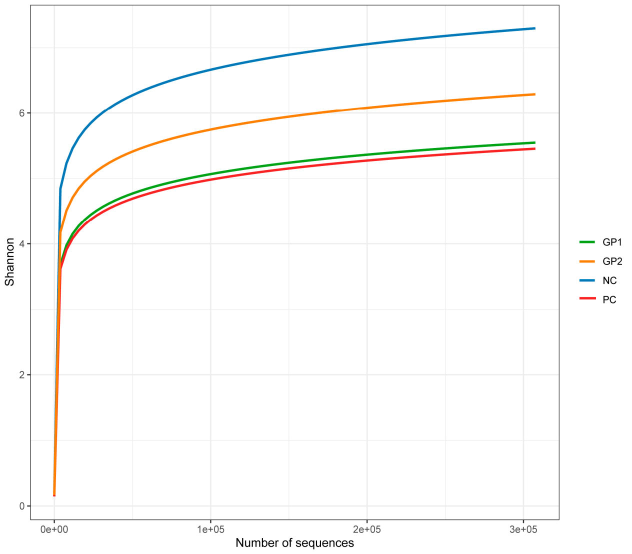
6
4
2
0
Shannon
0e+00
1e+05
2e+05
3e+05
Number of sequences
GP1
GP2
NC
PC

(B)

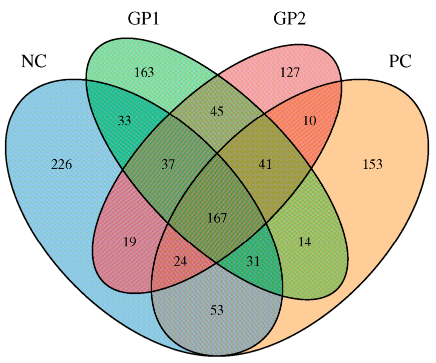

(C)

Figure S3. The OTU numbers (A), Shannon Rarefaction curves (B) and Venn diagram of OTU(C)

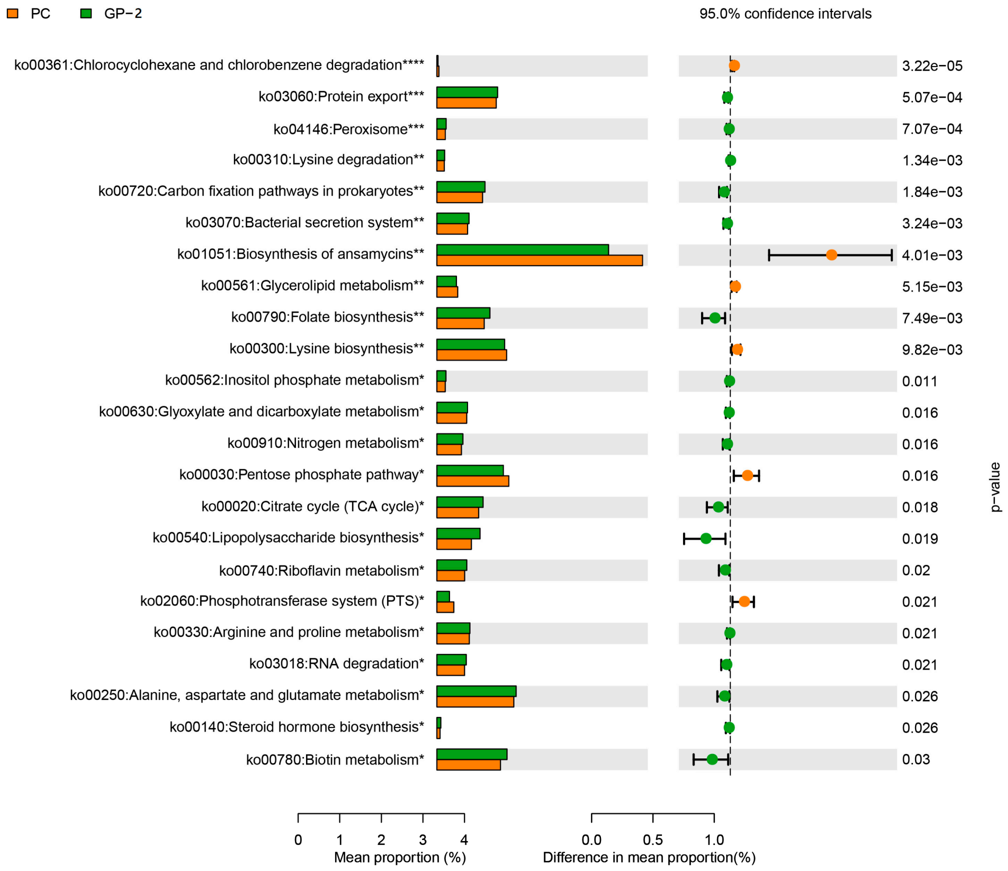

Figure. S4. Predicted and functions annotation was performed by PICRUSt. The supplement of GP-2 (300 mg/kg/day) on predicted functions in DSS-treated mice.
